# Ossifying Fibromyxoid Tumor of the Shoulder: A Case Report

**DOI:** 10.7759/cureus.63309

**Published:** 2024-06-27

**Authors:** Joel D Nash, Rohit Muralidhar, Abdullah Yousaf, Maria Castilla

**Affiliations:** 1 Medicine, Nova Southeastern University Dr. Kiran C. Patel College of Osteopathic Medicine, Fort Lauderdale, USA; 2 Medicine, William Carey University College of Osteopathic Medicine, Hattiesburg, USA; 3 General Surgery, HCA Florida Fawcett Hospital, Port Charlotte, USA

**Keywords:** s100-positive, painless subcutaneous mass, rare cancers, subcutaneous tumor, ossifying fibromyxoid

## Abstract

Ossifying fibromyxoid tumor (OFMT) is a rare, slow-growing, mesenchymal tumor with intermediate malignant potential, predominantly affecting middle-aged individuals. Histologically, it presents as a fibrous capsule or pseudocapsule, with a complete or incomplete lamellar bone shell surrounding oval/polygonal cells within a fibromyxoid matrix. Advances in immunohistochemistry have facilitated OFMT identification, with S100 protein expression and INI-1 loss being notable features. CD10 expression is also reported in a small minority of cases. Recent studies highlight a translocation of the PHF-1 gene, proposing a possible etiology for tumorigenesis. Treatment involves wide excision, with long-term follow-up for recurrence or metastasis.

In this case, a 61-year-old White male presented to the outpatient surgical office with a painless mass on his right shoulder. The patient reported that the mass first appeared three to four years prior and that it had been growing slowly since the initial presentation. On examination, the patient had a well-circumscribed, 1.5 x 1.5 cm, soft, nontender, nonmobile subcutaneous mass on his right shoulder. The mass was initially suspected to be a subcutaneous cyst based on physical exam, but surgical excision and histopathology established the diagnosis of OFMT that extended to the margins of the specimen. The patient underwent a wider excision for margins and has had a benign postoperative course. The patient was referred to dermatology and oncology for continuation of care.

This case demonstrates the necessity for a thorough work-up, appropriate excision, and histopathologic examination to rule in diagnoses of lower incidence with the potential for a worse prognosis. Appropriate and timely diagnoses can guide proper screening for cancer recurrence and management.

## Introduction

Ossifying fibromyxoid tumor (OFMT) is a rare mesenchymal tumor of undetermined cellular origin and intermediate malignant potential [1,2]. Since its discovery by Enzinger et al. in 1989 [3], there have been over 300 documented cases of OFMTs [4]. OFMTs are usually well-circumscribed, with a fibrous capsule or pseudocapsule and a partial or complete bone shell. The shell may be woven or lamellar bone [1,2,5,6]. Histologically, they consist of an inner core of oval/polygonal-shaped cells arranged in nests and cords within a bland fibromyxoid or hyaline matrix [1,2]. OFMTs can be classified as typical, atypical, or malignant, depending on their histological characteristics. A mitotic rate of >2/50 HPF is considered a hallmark feature of malignant OFMTs and is also a feature associated with atypical OFMTs. OFMT pathogenesis remains a mystery; however, recent studies have highlighted a translocation rearrangement of the gene PHF-1 on chromosome 6p21 in the majority (50-85%) of cases [5,7]. There is no definitive treatment algorithm for OFMTs currently, but expert recommendations include wide-based excision of the tumor with follow-up examinations for recurrence/metastasis screening [4,8]. This article presents a case of OFMT that was initially presumed to be a more benign diagnosis and underscores the need for clinicians to conduct a thorough work-up to rule out more insidious diagnoses.

## Case presentation

A 61-year-old White male presented to an outpatient surgical office with a painless mass on his right shoulder. The patient reported that the mass first appeared three to four years ago and had been growing slowly since its initial presentation. On physical examination, the patient was noted to have a well-circumscribed, 1.5 x 1.5 cm, soft, nontender, nonmobile, nonerythemic mass on his right shoulder. The patient's past medical history was significant for hypertension.

**Figure 1:**
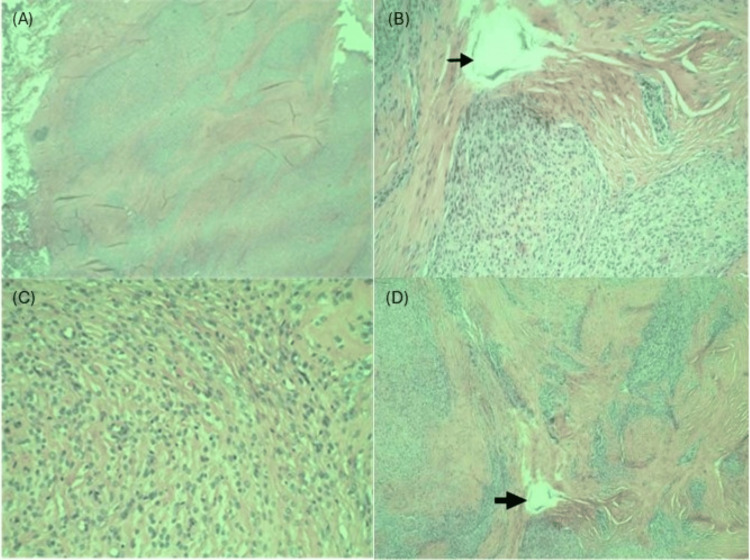
Histological examination of the excised ossifying fibromyxoid tumor with H&E staining (A) Low power (2x) view showing circumscribed, biphasic appearance with myxoid areas and fibrous septa. (B) High power (40x) view of focal ossification (arrow) within low cellularity fibrous septa. Myxoid areas with moderate cellularity are composed of uniform, bland ovoid to spindle-shaped cells. (C) High power (40x) view of the myxoid area with ovoid to spindle-shaped cells exhibiting low-grade nuclear features including small uniform nuclei and indistinct nucleoli. (D) Medium power (10x) view showing myxoid areas with moderate cellularity and low cellularity fibrous septa with focal ossification (arrow).

**Figure 2:**
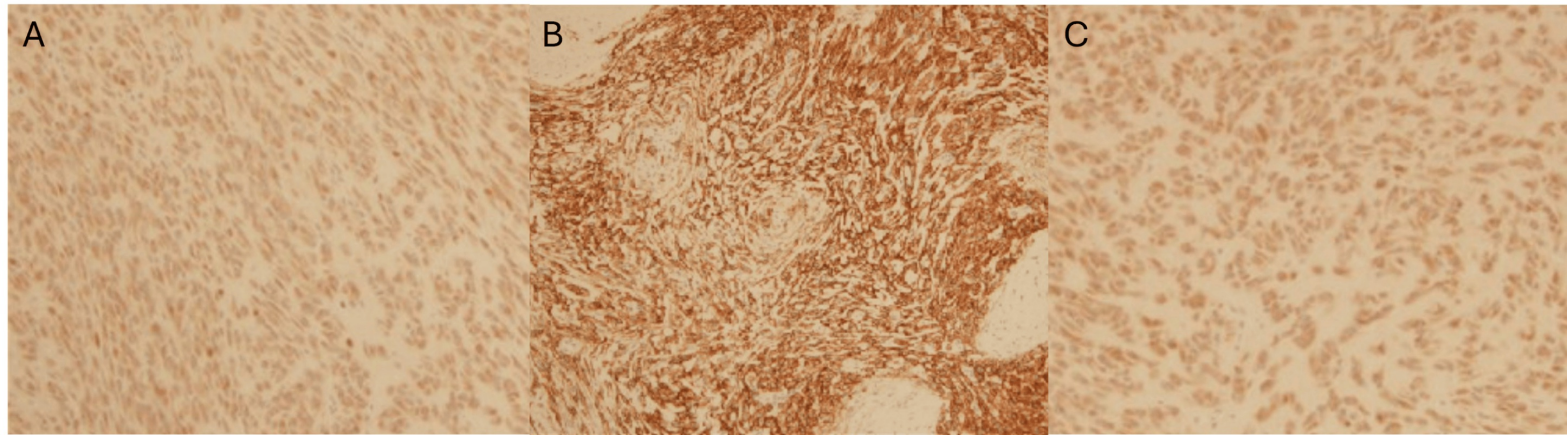
CD10, S100, and desmin immunohistochemical stains of excised subcutaneous mass A) Medium power (10x) view showing positive S100 immunohistochemical stain. B) Medium power (10x) view showing positive CD10 immunohistochemical stain. C) Medium power (10x) view showing positive desmin immunohistochemical stain.

The patient was suspected to have a sebaceous cyst due to the physical exam findings of a soft, non-tender, non-mobile, well-circumscribed superficial mass. The patient was scheduled for a surgical excision, during which the mass was excised under local anesthesia and sent to pathology for examination. The pathology report confirmed a 1.5 x 1.0 x 1.0 cm mass. Histological examination revealed uniform lobules and round to fusiform-shaped cells arranged in nests and cords, set in variable fibromyxoid stroma with fragments of lamellar bone at the center and periphery (Figure 1). Histological examination also revealed that mitotic figures were scant, and no necrosis was present. A diagnosis of typical OFMT was made. Immunohistochemistry testing of tumor cells was positive for S100, CD10, glial fibrillary acidic protein (GFAP), and desmin expression (Figure 2).

The pathology report also stated that the lesion extended into the inked margins of the excised specimen. The patient was subsequently rescheduled for a wide excision for margins and was referred to dermatology and oncology services for the establishment of care. The patient had a benign post-operative course and was not prescribed any additional medications. The patient was also scheduled for a one-year follow-up in the office for screening for recurrence.

## Discussion

OFMTs are rare neoplasms of unknown origin that are known to potentially recur and undergo malignant transformation. They have been reported in a wide age range of individuals from 10 to 88 years old [5], but the majority of tumors appear in middle-aged patients, around 50 years old, with a slightly higher incidence in males [2,5,9]. OFMTs are designated as either typical, atypical, or malignant based on nuclear grade, mitotic activity, and cellularity (cellular density) [4]. Typical OFMTs are characterized by low nuclear grade, low cellularity, and under two mitoses per 50 HPF, while malignant OFMTs are classified as high nuclear grade or high cellularity and greater than two mitoses per 50 HPF. Atypical OFMTs are described as histologically different from typical OFMTs, but it does not meet the aforementioned full criteria for malignant OFMTs [4,10]. Malignant OFMTs have a significantly higher risk for distant metastasis and recurrence than typical OFMTs [4].

Immunohistochemical markers

Advances in immunohistochemistry have been helpful in the identification of OFMTs, with S100 protein being expressed in roughly 75% of cases [1]. S100 protein has been associated more with typical OFMTs, with one study reporting 75% of typical OFMTs staining for S100, as opposed to 42% for malignant OFMTs. Desmin is the other protein frequently associated with OFMTs, estimated to stain in roughly 25% of tumors according to the most recent literature [1,2,5]. Loss of INI-1, a tumor suppressor gene, in a mosaic pattern, has also been documented in 74% of cases. Other immunohistochemical (IHC) markers less observed in OFMTs include smooth muscle actin (SMA), cytokeratins, epithelial membrane antigen, MUC4, and CD56 [2,5]. CD10 is also reported in less than 10% of cases [9]. For this reason, the case demonstrates a rare and unique pattern of immunohistochemical staining for OFMTs.

Pathogenesis and genetics

Despite tumorigenesis remaining unknown, there have been recent studies identifying a potentially significant translocation mutation of the PHF-1 gene on chromosome 6p21. The PHF-1 gene encodes for a protein controlling parts of embryonic stem cell differentiation through a downstream interaction with proteins encoded by the polycomb-repressive complex 2 (PRC2) gene, ultimately guiding histone methylation on DNA [5,7,11]. The frequency of this particular 6p21 gene translocation has been identified in 50 to 85% of OFMT cases per several studies, thus proposing a likely etiology to tumorigenesis. Further studies, however, are still needed to confirm its role in OFMTs. With this translocation being so prevalent, it opens the door for other genetic testing modalities such as fluorescence in situ hybridization (FISH) or RNA sequencing to aid in the diagnosis of OFMT cases that may be more histologically ambiguous [5,7].

Clinical features

OFMT has been described as a well-circumscribed, slow-growing, painless, subcutaneous mass that typically grows on the head, neck, trunk, and extremities. Other atypical sites, however, have recently been reported, including the oropharynx, chest wall, mediastinum, spine, intra/extracranially, and in the retroperitoneal cavity [2,4,8,12]. Imaging of OFMTs can be ambiguous, with radiography and CT of the lesion variably showing vague soft tissue masses with or without peripheral calcifications. MRI is also nonspecific and is reported to show a mass that is isointense to muscle on T1-weighted imaging and a hyperintense mass on T2-weighted imaging. Nuclear scans with technetium-99 may show increased uptake secondary to the lamellar bone formation within OFMTs. Because of the nonspecific physical manifestations of OFMTs, other differential diagnoses could include sclerosing epithelioid fibrosarcoma, malignant peripheral nerve sheath tumors, and ossifying hematomas [4], in addition to sebaceous cysts as in this case. 

Demographics and summary of current literature

To date, OFMT remains a rarely observed tumor, with over 300 reported cases. OFMTs statistically have been shown to have a slightly higher incidence in males, with one study reporting a male-to-female ratio of 1.5:1 [1]. OFMTs also have a median age of around 50 years old; however, it has been documented in a wide range of ages, from 14 to 83 years old [4].

Treatment

OFMTs are managed with wide-based excision of the mass followed by long-term patient follow-up for screening of tumor recurrence or distant metastasis [4,8]. There have been several documented cases of tumor recurrence years after surgical excision, which is the reason for long-term patient follow-up for screening of recurrence and/or metastasis [9]. There is very limited data mentioning the use of chemotherapy or radiation for any form of primary OFMTs. To date, the efficacy of chemotherapy or radiation is not well studied and therefore has not been universally accepted as a standard of care [5]. Only three case reports to date have mentioned the use of local radiation at the primary site of a malignant OFMT, with two of the patients reporting no recurrence after five and a half years and 18 months, respectively [13].

## Conclusions

This case underscores the critical need to recognize OFMTs and educate patients about their nature and recurrence risk. Accurate tumor identification and patient education are essential for targeted, personalized care. Educating patients about OFMT recurrence and the need to monitor for abnormal soft tissue masses may lead to quicker physician consultations and thus expedite screening for OFMT recurrence/metastasis. Despite being rare, OFMTs can be mistaken for other, more benign masses, as shown here. Clinicians must consider OFMTs in patients with subcutaneous masses, regardless of location or age. Incorporating this awareness into clinical practice can enhance diagnosis speed, management effectiveness, and cancer recurrence screening. Additionally, more research needs to be done on the prevalence of biomarkers such as CD10 for OFMT screening.
